# Prime-Boost Vaccination with SA-4-1BBL Costimulatory Molecule and Survivin Eradicates Lung Carcinoma in CD8^+^ T and NK Cell Dependent Manner

**DOI:** 10.1371/journal.pone.0048463

**Published:** 2012-11-08

**Authors:** Abhishek K. Srivastava, Rajesh K. Sharma, Esma S. Yolcu, Vahap Ulker, Kathryn MacLeod, Gunes Dinc, Haval Shirwan

**Affiliations:** 1 Institute for Cellular Therapeutics, Department of Microbiology and Immunology and James Brown Cancer Center, University of Louisville, Louisville, Kentucky, United States of America; 2 ApoVax Inc., Louisville, Kentucky, United States of America; Wayne State University, United States of America

## Abstract

Subunit vaccines containing universal tumor associated antigens (TAAs) present an attractive treatment modality for cancer primarily due to their safety and potential to generate long-term immunological responses that can safeguard against recurrences. However, TAA-based subunit vaccines require potent adjuvants for therapeutic efficacy. Using a novel form of the 4-1BBL costimulatory molecule, SA-4-1BBL, as the adjuvant of choice, we previously demonstrated that a single vaccination with survivin (SVN) as a *bona fide* self TAA was effective in eradicating weakly immunogenic 3LL tumors in >70% of C57BL/6 mice. The present study was designed to i) assess the therapeutic efficacy of a prime-boost vaccination and ii) investigate the mechanistic basis of vaccine efficacy. Our data shows that a prime-boost vaccination strategy was effective in eradicating 3LL lung carcinoma in 100% of mice. The vaccine efficacy was correlated with increased percentages of CD8^+^ T cells expressing IFN-γ as well as potent killing responses of both CD8^+^ T and NK cells in the absence of detectable antibodies to ssDNA as a sign of autoimmunity. Antibody depletion of CD8^+^ T cells one day before vaccination completely abrogated therapeutic efficacy, whereas depletion of CD4^+^ T cells had no effect. Importantly, NK cell depletion had a moderate (∼50% reduction), but significant (p<0.05) effect on vaccine efficacy. Taken together, these results shed light on the mechanistic basis of the SA-4-1BBL/SVN subunit vaccine formulation in a lung carcinoma model and demonstrate the robust therapeutic efficacy of the prime-boost immunization strategy with important clinical implications.

## Introduction

A large body of research over several decades has provided unequivocal evidence for the role of immunosurveillance in the control of cancer [1]. More importantly, a series of clinical studies have recently demonstrated that immune system can effectively be exploited for the control and/or eradication of cancer [2], providing not only proof-of-feasibility but also great opportunity for the development of innovative immune therapies. Among various immunotherapeutic approaches, cancer subunit vaccines based on tumor associated antigens (TAAs) are attractive primarily because of the low cost associated with production, ease of administration into patients, and unmatched safety profile. However, the therapeutic efficacy of subunit vaccines based on self TAAs is limited by low immunogenicity due to self-tolerance to such antigens and various immune evasion mechanisms employed by the progressing tumors [3], [4]. To overcome these limitations, TAA-based therapeutic vaccine formulations require the inclusion of potent adjuvants that not only generate robust innate and adaptive immune responses with long-term immunological memory, but also overcome various immune evasion mechanisms employed by the tumors [4]. Vaccine efficacy can further be improved by the choice of TAAs; antigens that are specifically expressed/upregulated in the tumor as well as involved in tumor progression and/or immune evasion mechanisms represent ideal choices.

We have recently hypothesized that TNF costimulatory ligand family can be utilized as adjuvant of choice for the development of therapeutic cancer vaccines and focused on the 4-1BBL molecule as the lead candidate. The choice of 4-1BBL molecule was primarily based on its pleiotropic effects on cells of innate, adaptive and regulatory immunity. Signaling via 4-1BB receptor has been shown to regulate various immune responses, and in particular plays a critical role for the survival/expansion of CD8^+^ T cells, acquisition of effector function, and long-term immunological memory that safeguards against tumor recurrences [5]–[8]. Inasmuch as 4-1BBL is a cell surface membranous protein and has no function in soluble form, recently we have generated a chimeric protein, from hereafter called as SA-4-1BBL, by fusing the extracellular functional domain of 4-1BBL to a modified form of core streptavidin (SA). This soluble SA-4-1BBL has shown potent immune activity as an adjuvant component of TAA-based vaccines by targeting various cells of innate, such as DCs and NK cells, and adaptive, such as CD4^+^ and CD8^+^ T cells, immunity in various preclinical tumor models [5]–[7], [9]. Most importantly, SA-4-1BBL also modulates regulatory immunity by reversing tumor induced clonal anergy, rendering T effector (Teff) cells resistant to suppression by CD4^+^CD25^+^FoxP3^+^ T regulatory (Treg) cells [7], and inhibiting the conversion of Teff cells into Treg cells through the production of IFN-γ [10].

Survivin (SVN) has significant potential as a universal TAA as it is overexpressed by various tumors and involved in multiple signaling mechanisms that regulate tumor cell survival, proliferative capacity, and secretion of various growth and angiogenic factors that help tumor progression [11]–[14]. The over-expression of SVN correlates with tumor progression and constitutes a poor prognostic factor for cancer patients [15], [16]. Importantly, CD8^+^ T cells responses to SVN have been identified in both cancer patients and in tumor-bearing mice [12], [17], [18], suggesting that immune tolerance to this self-protein is incomplete, which further validates the use of SVN as TAA for subunit vaccines against cancer. Consistent with this notion, SVN has been used for vaccination as a TAA in various cancer clinical trials with reported immunogenicity, but minimal clinical efficacy [19], [20]. Although the reason(s) for lack/minimal clinical efficacy of the SVN-based vaccines are not known, the absence of a potent adjuvant as the component of vaccine formulations may provide an explanation.

We have recently demonstrated that a single vaccination with SVN containing SA-4-1BBL as adjuvant eradicates 3LL tumors in >70% of mice in a therapeutic setting [7]. The main objective of the present study was to test if a prime-boost immunization strategy further improves SA-4-1BBL/SVN vaccine efficacy and elucidate its mechanistic basis with particular focus on CD4^+^ T, CD8^+^ T, and NK cells as the primary cellular targets for SA-4-1BBL. We herein report that a prime-boost vaccination regimen with SA-4-1BBL/SVN was effective in eradicating 3LL tumor in all mice. Vaccine efficacy was totally dependent on CD8^+^ T cells, NK cells played a moderate, but significant role, and CD4^+^ T cells had no detectable effect under the setting tested. Taken together, these results illuminate the mechanistic basis of SA-4-1BBL/SVN vaccine formulation and demonstrate its robust therapeutic efficacy in a prime-boost setting with important clinical implications.

## Results

### Expression and purification of recombinant SVN

**Figure 1:**
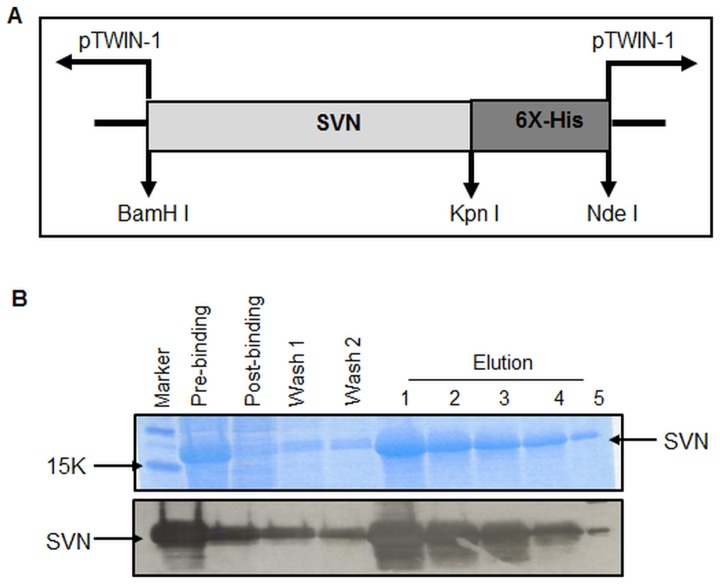
Production of recombinant mouse SVN. (A) Schematic representation of pTWIN-1 expression vector with mouse SVN cDNA. (B) Purification of SVN protein from inclusion bodies using IMAC as assessed by SDS-PAGE gels stained with Coomassie blue (top) or Western blot (bottom).

A cDNA encoding the complete mouse SVN engineered to include 6X-His residues at C-terminus (Fig. 1A) was expressed in *E. coli* using the pTWIN-1 expression vector. Inclusion bodies were isolated, denatured with Guanidine-HCl, and purified using IMAC taking advantage of the 6X-His tag. All purification steps were performed in the presence of 10 µM ZnCl_2_ and 5 mM β-ME to assist with proper folding and reduce oligomerization. The purified protein runs approximately as a 16.5 kDa band on SDS-PAGE (Fig. 1B top panel) and reacts with SVN antibody when analyzed on western blots (Fig. 1B bottom panel).

### A prime-boost immunization scheme with SA-4-1BBL/SVN is effective in eradicating 3LL tumors in all mice

**Figure 2:**
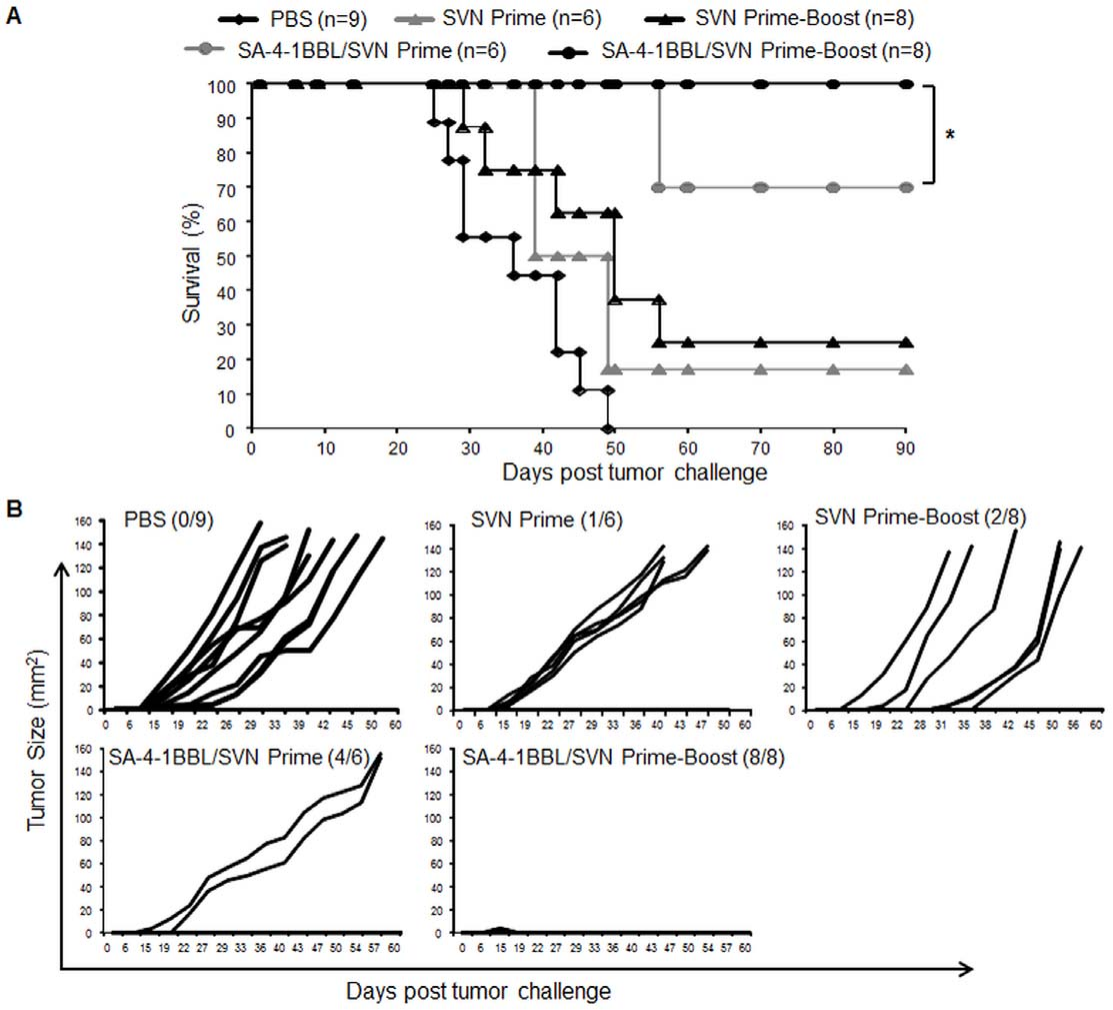
Prime-boost vaccination with SA-4-1BBL/SVN completely eradicates the 3LL tumors. (A) C57BL/6 mice were challenged with live 1×10^5^ 3LL tumor cells and vaccinated s.c. with SVN (50 µg) alone or SVN admixed with SA-4-1BBL (25 µg) once on day 6 or twice on day 6 and day 11 post-tumor challenge. (B) Tumor progression for individual mice shown in (A). *P<0.05 for SA-4-1BBL/SVN prime-boost vs. SA-4-1BBL/SVN prime setting.

We have previously demonstrated that a single vaccination with SA-4-1BBL/SVN was effective in eliminating 3LL tumors in >70% of C57BL/6 mice [7]. We herein asked if a prime-boost immunization scheme further improves the efficacy of the vaccine. C57BL/6 mice were challenged s.c. with 1×10^5^ live 3LL tumor cells and vaccinated six days later once with SVN protein alone (50 µg) or admixed with SA-4-1BBL (25 µg). As shown in Fig. 2, single vaccination with SA-4-1BBL/SVN was effective in eradicating 3LL tumors in 70% of the mice. Importantly, this efficacy was improved to 100% when a booster injection of SA-4-1BBL/SVN was given 5 days following primary immunization. This potent therapeutic efficacy was due to SA-4-1BBL as prime-boost-immunization with SVN alone was effective in eradicating tumors in only 25% of the mice (Fig. 2). Taken together, these data confirm our previous findings with a single vaccination [7] and demonstrate the robust therapeutic efficacy of prime-boost immunization with SA-4-1BBL/SVN vaccine formulation.

### The therapeutic efficacy of the vaccine is associated with robust CD8^+^ T cells and NK cells effector responses

**Figure 3:**
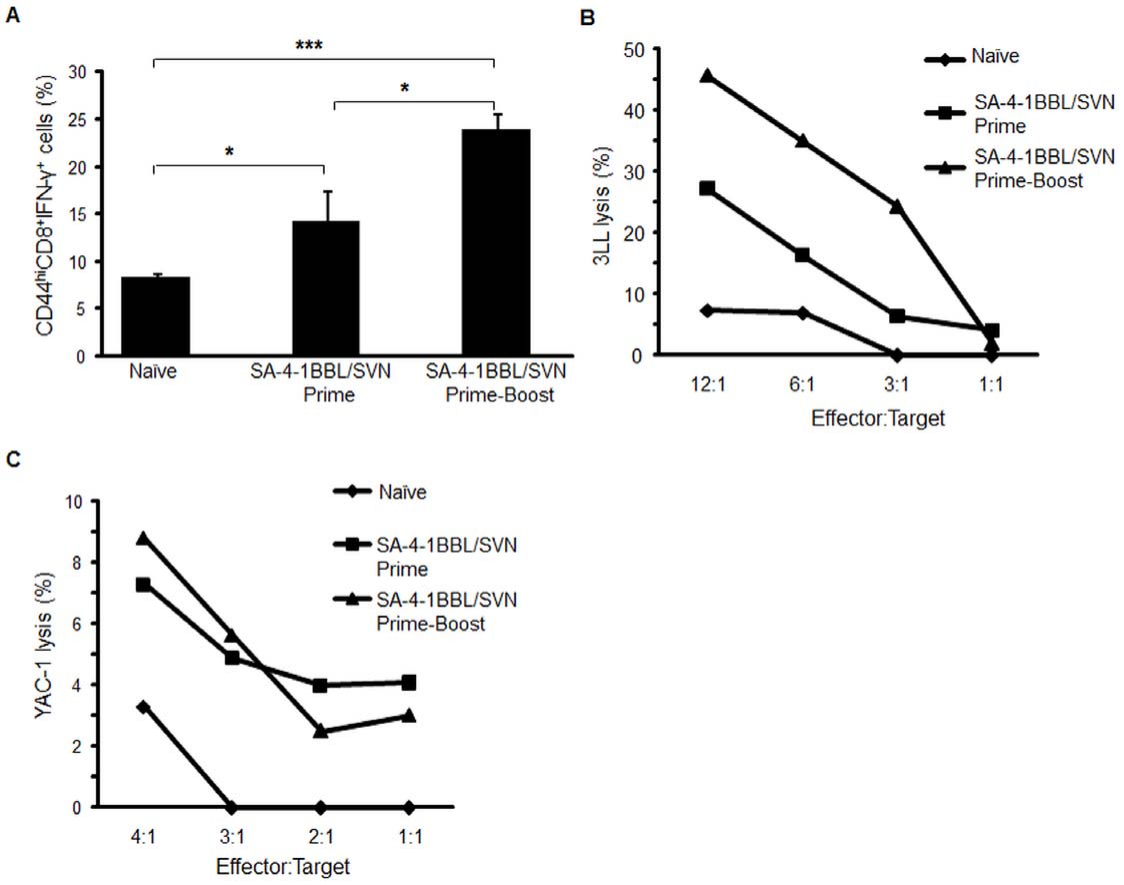
Prime-boost vaccination with SA-4-1BBL/SVN induces strong anti-tumor CD8^+^ T cell and NK cell effector responses. Mice in the prime and prime-boost groups that have undergone effective immunotherapy were boosted with the same SA-4-1BBL/SVN vaccine formulation (>90 days post-tumor challenge). (A) Vaccine-draining lymph nodes were harvested 7 days later and assessed for the frequency of CD44^+^CD8^+^IFN-γ^+^ T cells, while (B) splenocytes from the same animals were used for CTL killing of 3LL tumor cells, and (C) NK killing of YAC-1 tumor cells. *P* values were calculated using student t-test.

To evaluate the mechanistic insight into the vaccine efficacy, we evaluated the status of IFN-γ in the long-term tumor free animals one week after boosting with SA-4-1BBL/SVN formulation. As shown in Fig. 3A, long-term mice subjected to prime-boost vaccination with SA-4-1BBL/SVN formulation had significantly enhanced frequency of CD8^+^ T cells expressing IFN-γ, which translated into higher cytotoxicity against 3LL tumors *in vitro* (Fig. 3B) as compared to naïve and mice with primary immunization only. Importantly, long-term mice with effective immunotherapy also had heightened NK cell killing response against YAC-1 tumor targets, however this response was comparable for both prime only and prime-boost groups (Fig. 3C). Collectively, these data demonstrate that SA-4-1BBL serves as an effective adjuvant component of the vaccine formulation for the generation of both CD8^+^ T and NK cell effector responses.

### CD8^+^ T cells play an obligatory role while NK cells play a moderate, but significant, role in the therapeutic efficacy of the vaccine

**Figure 4:**
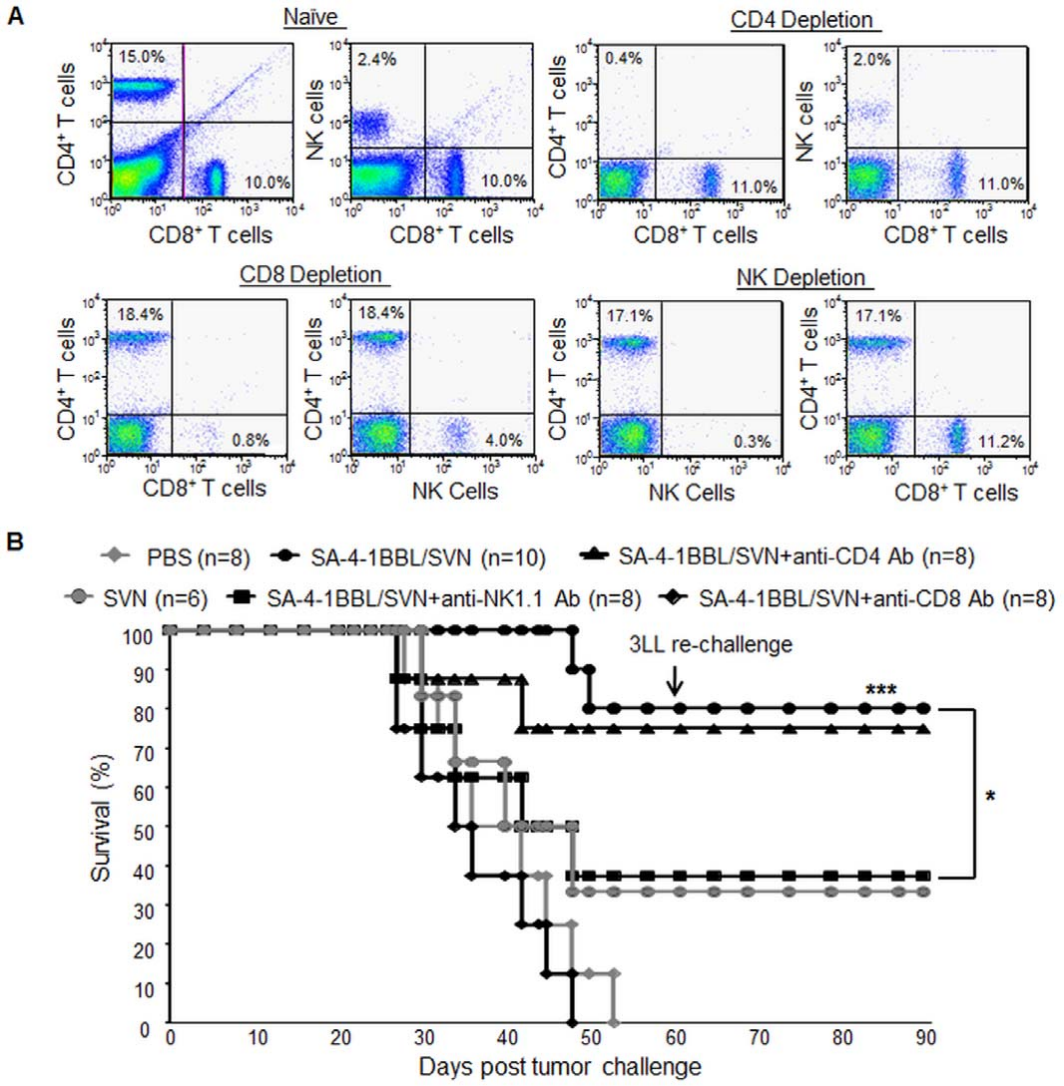
Therapeutic efficacy of SA-4-1BBL/SVN vaccine requires CD8^+^ T cells with a significant contribution from NK cells. C57BL/6 mice challenged with live 1×10^5^ 3LL tumor cells were treated i.p. with depleting doses (500 µg/Ab/mouse) of Abs against CD8, CD4 and NK1.1 molecules one day before vaccination with SA-4-1BBL/SVN. (A) Specific depletion of the targeted cell populations. (B) Effect of depletion of various cell populations on vaccine therapeutic efficacy. To assess anti-tumor immune memory, long-term survivors in the SA-4-1BBL/SVN group were inoculated with a second dose of live 3LL tumor cells on day 60 post initial tumor-challenge and monitored for an additional 30 days. *P<0.05, SA-4-1BBL/SVN vs. NK1.1 treated SA-4-1BBL/SVN group; ***P<0.001, SA-4-1BBL/SVN *vs.* CD8 Ab treated/SA-4-1BBL/SVN or PBS groups.

Our observations correlating vaccine therapeutic efficacy against 3LL tumors to heightened T and NK cell effector responses led us to directly test the role of these cell populations in vaccine therapeutic efficacy. We chose one vaccination as a more sensitive setting for this purpose. C57BL/6 mice were treated *in vivo* with Abs against CD4, CD8, and NK1.1 molecules one day prior to vaccination with SA-4-1BBL/SVN. This treatment resulted in complete and specific depletion of the targeted cell populations (Fig. 4A). The depletion of CD8^+^ T cells completely abrogated the efficacy of the vaccine as all mice treated with anti-CD8 Ab expired from tumor burden within 50 days of tumor inoculation. In marked contrast, depletion of CD4^+^ T cells had no significant effect (∼8% reduction) while depletion of NK cells resulted in moderate, but significant (p<0.05) reduction (∼50%), in vaccine efficacy (Fig. 4B).

We also noticed that one vaccination with SA-4-1BBL/SVN was effective in eradicating 3LL tumor in 80% of the mice (Fig. 4B) *vs.* 70% observed in our previously published studies [7] and those presented in Fig. 2A. This difference is not significant and may be due to interexperimental variations. To assess the persistence of immune memory to 3LL tumor, long-term tumor free mice that had undergone effective therapy were re-challenged with live 1×10^5^ 3LL tumor cells 60 days post initial tumor inoculation. Impressively, none of these mice developed tumor in a course of 90 days (Fig. 4B), demonstrating long-term and protective immune memory that may safeguards against recurrences.

### The therapeutic efficacy of SA-4-1BBL/SVN vaccine is not associated with detectable autoantibodies to ssDNA

**Figure 5:**
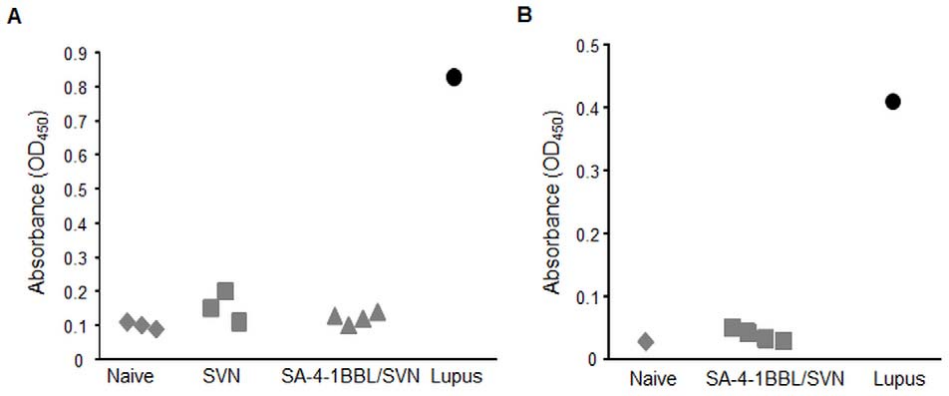
Vaccination with SA-4-1BBL/SVN does not induce autoantibodies against ssDNA. (A) Long-term (>60 days) mice that had eliminated 3LL tumor in response to one vaccination (prime) were boosted with the same dose of SA-4-BBL/SVN vaccine. Serum was collected 7 days later and assessed for the titers of autoantibodies against ss DNA. (B) Naïve mice without tumor challenge were vaccinated three times on days 0, 5, and 31 and serum was collected on day 38 to assess the titers of antibodies against ssDNA. Individual (A) or pooled serum (B) from naïve mice was used as background control while lupus serum was used as positive control in a standard ssDNA ELISA.

Inasmuch as SVN is a *bona fide* self TAA, we next assessed if vaccination with SA-4-1BBL/SVN results in the generation of autoantibodies against ssDNA as a sign of systemic autoimmunity [21]. Serum harvested from long-term mice (>60 days) that had undergone effective immunotherapy due to vaccination with SVN alone or SA-4-1BBL/SVN was tested for the presence of Abs against ssDNA using a standard ELISA [21]. As shown in Fig. 5A, we did not detect significant levels of anti-ssDNA Abs in the serum of mice with successful immunotherapy as compared with serum from naïve controls. To eliminate the impact of tumor on the generation of autoantibodies against ssDNA, mice without tumor challenge were vaccinated 3 times with SA-4-1BBL/SVN. Serum harvested from these mice also scored negative for Abs to ssDNA (Fig. 5B). In contrast, there was significant titers of Abs against ssDNA in the serum of lupus inflicted mice. Taken together, these data demonstrate that vaccination with SA-4-1BBL/SVN achieves therapeutic efficacy against 3LL tumors in the absence of systemic autoimmunity as assessed by autoantibody titers against ssDNA.

## Discussion

In the present study, we tested the therapeutic efficacy of SA-4-1BBL/SVN vaccine formulation in a prime-boost immunization setting against 3LL lung carcinoma and shed light on the possible mechanistic basis of vaccine efficacy. Prime-boost vaccination had robust therapeutic efficacy resulting in eradication of 3LL tumor in all mice without detectable toxicity. CD8^+^ T cells were required for therapeutic efficacy, while NK cells played a moderate, but significant role.

The therapeutic efficacy of TAA-based subunit vaccines depends on both the nature of TAAs and potency of the adjuvants used in the vaccine formulation. Antigens that not only mark the tumor for immune destruction, but also play a role in tumor survival/progression and/or immune evasion are ideal candidates as they are not subjected to antigenic drift and reduce the chances of escape by edited resistant tumors from antigenic variants. Similarly, adjuvants that increase the intensity and breadth of the immune response against the TAA and overcome immune evasion mechanisms employed by the progressing tumor stand the best chance to succeed in a therapeutic setting. Both SVN, as a universal TAA involved in tumor survival/progression [11], [14], and SA-4-1BBL, as an adjuvant having pleiotropic effects on innate, adaptive, and regulatory immune responses, [5]–[7] meet the aforementioned criteria.

SVN has been extensively used as a TAA for cancer immunotherapy primarily due to its high level of expression in most cancers, lack/minimal expression in normal tissues, and involvement in tumor survival, progression, and chemoresistance [22]. However, vaccines based on SVN as TAA have shown limited therapeutic efficacy in preclinical models [23], [24] and no therapeutic, but limited immunogenic efficacy in the clinic [19], [20], [25]. Although there are various factors that may affect the limited efficacy of SVN based vaccines, the lack of an adjuvant with potent immune stimulatory activity along with the ability to concomitantly overcome various immune evasion mechanisms employed by the progressing tumor may provide a potential explanation. Consistent with this notion, we have recently demonstrated that SA-4-1BBL has the aforementioned features of a desired adjuvant since a single vaccination with SA-4-1BBL/SVN resulted in the eradication of 3LL tumors in >70% of mice [7]. We herein demonstrated that a prime-boost vaccination strategy can further improve vaccine efficacy, resulting in eradication of 3LL tumors in all mice. This finding is highly significant in prospects of translational scope of this vaccine into clinical trials as more than one vaccine treatment is often required to achieve clinical efficacy. The therapeutic efficacy of the vaccine was contingent on CD8^+^ T cells as the depletion of this cell population one day before vaccination resulted in tumor growth in all mice. These findings are consistent with studies demonstrating that vaccination with SVN generates vigorous cytotoxic responses both *in vitro* and *in vivo* [12], [17], [18]. A DNA-based vaccine encoding SVN as TAA and NKGD2 as an immune stimulator generated both NK and CD8^+^ T cell effector responses that resulted in protection against carcinomas [26]. Similarly, another DNA-based vaccine encoding SVN and chemokine CCL21 was shown to inhibit angiogenesis and induced potent CD8^+^ T cell and IFN-γ responses against Lewis lung carcinoma with significant prophylactic and therapeutic efficacy [27]. SA-4-1BBL/SVN vaccination also enhanced the memory response evaluated by the no tumor development on the second lethal tumor challenge on day 60 in the long-term animals suggested that vaccine has potential to control recurrences.

The therapeutic efficacy of SA-4-1BBL/SVN vaccine also involved NK cells. Mice that underwent effective therapy had significant NK cell *in vitro* killing activity against YAC-1 tumor cells. Importantly, the Ab depletion of NK cells one day before vaccination reduced the therapeutic efficacy of the vaccine from 80% to 40%, providing direct evidence for the involvement of these cells in tumor eradication. These findings are in the agreement and confirming our previously published study demonstrating that SA-4-1BBL as the adjuvant component of HPV E7 subunit vaccine generated NK cell activity that significantly contributed to the therapeutic efficacy of the vaccine in a cervical cancer mouse model [6]. We (Srivastava *et al.*, unpublished observations) and others [28], [29] have demonstrated that 3LL tumor cells down-regulate the cell surface expression of MHC class I molecules as a means of escaping CD8^+^ T cell mediated killing. Therefore, the ability of SA-4-1BBL to induce both CD8^+^ T cells that recognize and destroy tumor in the context of MHC class I molecules and NK cells that destroy tumor cells lacking MHC class I serves as a potent means of eradicating tumor. The crucial role of NK cells in killing MHC class I negative tumors have been previously demonstrated [30]. Inasmuch as IFN-γ is a potent inducer of MHC class I molecules [31], it is tempting to speculate that the moderate role of NK cells, as compared with CD8^+^ T cells, in the therapeutic efficacy of SA-4-1BBL/SVN against the 3LL tumors is due to the ability of the vaccine to induce a strong IFN-γ response. Particularly, intratumoral expression of IFN-γ by CD8^+^ T cells may upregulate the expression of MHC class I molecules on tumor cells, thereby making them better targets for CD8^+^ T cell killing. In addition, NK cells and CD8^+^ T cells can also regulate the activity of each other. There could be a direct or indirect crosstalk between these two cell populations. Activated NK cells are reported to induce the adaptive immune response via IFN-γ production which promotes the Th1 polarization of antigen specific T cells [32]. On the other hand, antigen specific T cells can directly activate NK cells by producing IL-2 [33]. The crosstalk between these two effector cells against cancer may have provided the immune system the well sought edge to overcome tumor-induced immune suppression, which in turn translated into tumor elimination observed with SA-4-1BBL/SVN vaccine.

CD8^+^ T cells represent a critical effector mechanism of tumor eradication using various immunotherapeutic approaches [34]–[36]. Although cross-presentation of exogenous antigens by DCs plays a critical role in CD8^+^ T cell activation and effector function as we have recently demonstrated using a SA-4-1BBL-based vaccine [5], other non-professional antigen presenting cells conditioned by cytokines within the tumor microenvironment may also activate CD8^+^ T cells. In particular, a recent study has demonstrated adoptive transfer of tumor-specific CD8^+^ T cells engineered to secrete IL-12 improved their therapeutic efficacy against melanoma [37]. The observed therapeutic effect was due to IL-12 sensitizing not only tumor infiltrating DCs and macrophages, but also myeloid-derived suppressor cells for CD8^+^ T cells activation and effector function [37]. This reprogramming of myeloid-derived suppressor cells for antigen presentation was partially dependent on IFN-γ. Therefore, it is likely that in addition to serving as a costimulatory molecule for direct activation of CD8^+^ T cells, SA-4-1BBL may also alter the function of myeloid-derived suppressor cells either through a direct effect or indirectly via IFN-γ. Future studies will address this issue.

The depletion of CD4^+^ T cells one day before immunization with SA-4-1BBL/SVN did not significantly alter the therapeutic efficacy of the vaccine. Although the role of CD4^+^ T cells is well established for the induction and maintenance of memory CD8^+^ T cells [38], our data show that in this present setting CD4^+^ T cells are not required for SA-4-1BBL/SVN vaccine efficacy. This may be due to the preferential and potent function of SA-4-1BBL in driving CD8^+^ T cell responses [5], [7], [9]. Our observation is consistent with a study demonstrating that potent adjuvants, such as a combination of agonists for CD40 costimulatory receptor and TLRs, can bypass the need for CD4^+^ T cell help for CD8^+^ T cell responses [39]. Alternatively, anti-CD4 Ab used to deplete CD4^+^ T cells will also eliminate CD4^+^CD25^+^Foxp3^+^ Treg cells, which have been shown to play a critical role in tumor-mediated tolerance of Teff cells [3]. Therefore, it is plausible that the elimination of Treg cells using the anti-CD4 Ab may counterbalance the negative effect of CD4^+^ Teff cell depletion on vaccine therapeutic efficacy. Selective depletion of Treg cells may address this issue, but lack of such available reagents leave this question open ended. Consistent with this notion, depletion of CD4^+^ T cells has been shown to improve vaccine therapeutic efficacy in several preclinical tumor settings [40], [41].

Inasmuch as SVN is a *bona fide* self TAA, we also assessed and did not find the generation of Abs against ssDNA by SA-4-1BBL/SVN vaccination in mice with and without tumor challenge as a sign of systemic autoimmunity. Importantly, we did not detect any visual signs of acute toxicity in vaccinated mice based on animal weight loss, unexpected mortality, gross anatomy, and macroscopic analysis of body organs, demonstrating the safety profile of the vaccine. The lack of acute toxicity is consistent with our previously published studies demonstrating that treatment of mice with 4-fold higher SA-4-1BBL over the therapeutic dose used in this study did not result in detectable toxicity [5], [7], [9].

Collectively, these results demonstrate that prime-boost vaccination with SA-4-1BBL/SVN generates potent CD8^+^ T and NK cell responses that translate into robust therapeutic efficacy against 3LL lung carcinomas. Therefore, these observations justify further development of SA-4-1BBL as adjuvant component of therapeutic tumor vaccines with significant clinical potential.

## Materials and Methods

### Mice and cell lines

C57BL/6 mice were purchased from The Jackson Laboratory, Taconic, or bred in our barrier animal facility at the University of Louisville. 3LL cell line was purchased from American Type Culture Collection (Manassas, VA).

### Cloning, expression, and purification of recombinant SVN

Mouse SVN cDNA was subcloned into the pTWIN-1-6X-His bacterial expression vector and expressed in C2566 *E. coli* cells (New England Biolabs, Ipswich, MA). Briefly, IPTG treated cells were harvested 3 h after induction, pelleted, and resuspended in 100 ml of lysis buffer (20 mM Tris, pH 7.0, 500 mM NaCl, 5 mM imidazole, 5 mM β-ME, 10 µM ZnCl_2_). Cells were lysed by ultrasonication and inclusion bodies were pelleted at 10,000×g for 10 min. Pellet was washed three times by resuspending in lysis buffer containing 1% Triton X-100, rotating 30 min at room temperature, and repelleting at 35,000×g for 30 min. The final pellet was resuspended in 100 ml lysis buffer containing 6 M Guanidine-HCl and rotated at RT overnight to solubilize inclusion bodies. After, centrifugation at 35,000× *g* for 30 min at 4°C to remove the insoluble material, supernatant was collected for IMAC purification using Talon® cobalt resin according to the manufacturer's instructions (Clontech Laboratories, Mountain View, CA) with the exception of including 0.1% Triton X-114 to remove endotoxin. All purification steps were performed in the presence of 10 µM ZnCl_2_ and 5 mM β-ME to assist with proper folding and reduce oligomerization. Protein was dialyzed against PBS, concentrated using an Amicon Ultra MWCO 10,000, and sterile filtered using a 0.22 µm filtration device. Protein concentration was measured using BCA and Bradford methods (Pierce). Construction, expression, purification, and characterization of SA-4-1BBL have been previously described [5]. All the proteins had minimal/undetectable levels of endotoxin (SVN = 0.066 EU/µg protein, SA-4-1BBL = 0.004 EU/µg protein).

### Tumor model and immunizations

C57BL/6 mice were inoculated s.c. with 1×10^5^ live 3LL tumor cells into the right flank and vaccinated s.c. on day 6 post-tumor challenge with SVN protein (50 µg) alone or mixed with SA-4-1BBL (25 µg). Mice were boosted with the same vaccine formulations 5 days post-priming and monitored for tumor growth. Tumor size was measured with caliper twice weekly as described [5]. The survival end-point was defined as tumor size of 12 mm in diameter, grade 3 tumor ulceration, or when mice showed signs of poor conditions (hunched posture, anorexia, dehydration, weight loss, inactivity, difficulty walking, dyspnea, or moribund). Tumor immune memory response in the SA-4-1BBL/SVN vaccination group was assessed by challenging long-term survivors with live 1×10^5^ 3LL tumor cells 60 days post initial tumor challenge. These mice were then monitored for tumor development for an additional 30 days. We also immunized a separate group of mice without tumor challenge subcutaneously with SVN (50 µg) admixed with SA-4-1BBL (25 µg) on days 0, 5, and 31 and euthanized the mice on day 38 to assess serum for the titers of antibodies against ssDNA as a sign of autoimmunity.

Depletion studies were performed by treating mice i.p. one day before vaccination using 500 µg/Ab/animal against mouse CD8 (clone 53.6.72), NK1.1 (clone PK 136), and CD4 (clone GK1.5) molecules. All antibodies were purchased from BioXCell (West Lebanon, NH). The depletion of individual cell type was assessed 5 days post Ab-treatment using flow cytometry and was found to be complete (>98%).

### Intracellular cytokine assay

For intracellular cytokine staining, lymph node cells were resuspended in MLR medium at 2×10^6^/ml and stimulated with PMA (5 ng/ml, Sigma) and ionomycin (500 ng/ml, Sigma) for 2 hrs at 37°C in a 5% CO_2_ incubator. GolgiPlug (1 µl/ml, BD PharMingen) was added to the activation mixture and cells were incubated for an additional 4 hrs. Cells were stained with anti-CD44-APC Ab and anti-CD8-APC-Cy7 Ab, fixed with 4% paraformaldehyde, followed by staining using anti-IFN-γ-PE-Cy7 Ab or isotype control Abs. FACS Caliber or LSR-II (BD Biosciences) was used for cell acquisition followed by analysis using CellQuest (BD Biosciences), FlowJo (Tree Star), and Diva (BD Biosciences) software.

### *In vitro* CTL and NK cell killing assays

For *in vitro* cytotoxicity assay, long-term tumor free mice in SVN alone and SA-4-1BBL/SVN vaccination groups were boosted with the same vaccine formulations. Splenocytes from the boosted mice were harvested 7 days later and incubated in complete MLR medium supplemented with 10 µg SVN protein/ml and 50 IU/ml of IL-2. Five days post culturing, viable lymphocytes were recovered by centrifugation over a Ficoll gradient and used as effectors against 3LL tumor targets using JAM assay for CTL killing and YAC-1 tumor targets for NK cell killing as previously described [6].

### Detection of autoantibodies against ssDNA

Autoantibodies against ssDNA, as indicators of systemic autoimmunity [21], were assessed using ELISA. Briefly, 96-titers flat bottom plates were coated with 1 µg/well of heat-denatured calf thymus DNA (ssDNA, Sigma) at 4°C overnight. Wells were washed twice with washing buffer containing Borate Buffered Saline (BBS) supplemented with 0.5% Tween 20 followed by incubation in blocking buffer containing BBS, 5% BSA , and 0.5% Tween 20 at 4°C for 1 hr. After washing twice, wells were incubated with various dilutions of serum harvested from mice vaccinated with SA-4-1BBL/SVN followed by incubation at 4°C overnight. Wells were washed 3 times, incubated with anti-mouse IgG-HRP at 4°C for 2 hr followed by incubation in freshly prepared substrate solution for 20 min. The optical density at 450 nm was measured using a spectrometer. Pooled serum from 3 lupus inflicted mice was used as a positive control.

### Statistical analysis

Statistical analyses were performed using the One-Way ANOVA and log rank test using SPSS software. *P*<0.05 and 0.001 were considered significant (*) and very significant (***), respectively.

### Ethics Statement

This study was approved by the University of Louisville Institutional Animal Care and Use Committee (IACUC) (Protocol number: 08142) and carried out in strict accordance with the recommendations in the Guide for the Care and Use of Laboratory Animals of the National Institutes of Health.
